# Further Exploration of the Psychometric Properties of GamTest: A Rasch Analysis

**DOI:** 10.3390/ijerph18094824

**Published:** 2021-04-30

**Authors:** David Forsström, Alexander Rozental, Anders Kottorp, Philip Lindner, Markus Jansson-Fröjmark, Hugo Hesser

**Affiliations:** 1 Department of Psychology, Stockholm University, Frescati Hagväg 8, 105 90 Stockholm, Sweden; 2 Centre for Psychiatry Research, Department of Clinical Neuroscience, Karolinska Institutet & Stockholm Health Care Services, Region Stockholm, Norra Stationsgatan 69, 113 64 Stockholm, Sweden; alexander.rozental@ki.se (A.R.); philip.lindner@ki.se (P.L.); markus.jansson-frojmark@ki.se (M.J.-F.); 3 UCL, Great Ormond Street, Institute of Child Health, London WC1N 1EH, UK; 4 Faculty of Health and Society, Malmö University, Jan Waldenströms Gata 25, 214 28 Malmö, Sweden; anders.kottorp@mau.se; 5 Centre for Dependency Disorders, Stockholm Health Care Services, 104 31 Stockholm, Sweden; 6 School of Law, Psychology and Social Work, Örebro University, 701 82 Örebro, Sweden; hugo.hesser@oru.se; 7 Department of Behavioural Sciences and Learning, Linköping University, 581 83 Linköping, Sweden

**Keywords:** gambling, negative consequences, GamTest, Rasch analysis, Playscan

## Abstract

GamTest is a self-rating scale of negative consequences of gambling, included in the popular responsible gambling tool Playscan as part of an overall risk assessment and feedback feature. Two previous psychometric evaluations of this instrument yielded contradictory results: in an online high-gambling population, a five-factor model was supported and the instrument had overall good psychometric properties, but in a low-gambling population, the same factor structure was not supported. Because GamTest is used with both low- and high-gambling populations, more psychometric research is needed to fully understand how the instrument works. The current study examined, for the first time, psychometric performance among a sample of low-gambling respondents using a Rasch analysis. Results indicated that the instrument could be improved by decreasing the scale-steps and removing several problematic items demonstrating misfit. Furthermore, the findings indicated that some items functioned differently depending on gender, and that a shortened, improved nine-item version could not differentiate between different levels of risk. Our findings suggest that the instrument would arguably benefit from being adapted for use in a low-gambling population.

## 1. Introduction

Using self-rated instruments to cover different aspects of problem gambling is a validated approach to determine the level of negative consequences for recreational, at-risk, and problem gamblers, in particular in online gambling settings. Several such instruments have been published and are in use in different contexts, among them the South Oaks Gambling Screen (SOGS) [1], the Lie/Bet Questionnaire [2], the NORC Diagnostic Screen for Gambling Problems [3], and the Problem Gambling Severity Index (PGSI) [4,5].

However, most well-established instruments were designed for screening rather than full risk assessment, and/or do not cover all the different facets of the construct of ‘problem gambling’ and its inherent negative consequences. A more recently developed instrument, GamTest [6,7], was designed to address these limitations. The GamTest is already used by several Nordic and European gambling providers, and is integrated in the popular responsible gambling (RG) tool Playscan. In Sweden alone, about 100,000 tests are carried out per annum. Past studies have highlighted the significance of the GamTest for gamblers who use Playscan [8,9,10]. However, it has been observed that many Playscan users begin but do not complete the GamTest [8]. Understanding the GamTest’s characteristics and psychometric properties is, therefore, critical in order to identify limitations and make adjustments to improve performance and thereby increase response rates.

Using data from a high-gambling population, the authors of [7] carried out the first psychometric evaluation of the GamTest, revealing a five-factor structure and overall good psychometric properties in terms of validity and reliability. The study did not, however, discuss the GamTest’s psychometric properties in relation to the general use of Playscan. Additionally, the GamTest’s psychometric properties for a general population that likely includes a large proportion of recreational, non-problem gamblers was not explored in the study. If the GamTest is to be used as a comprehensive risk assessment tool, it must cover the entire spectrum of gambling and its associated negative consequences, as well as be able to differentiate between different levels of risk. A second study was, therefore, performed to investigate the GamTest’s psychometric properties in an overall low-gambling population. The study found an inconclusive fit for the five-factor solution, but the instrument still showed good internal reliability [6]. The two studies highlighted the need for further psychometric evaluations using a modern psychometric test theory, including examining how well the instrument works in terms of its scale-steps, item functioning, and ability to differentiate between respondents with different risk profiles.

The overall aim was to further explore the accuracy of the GamTest as a way of measuring negative consequences associated with gambling, and identify suitable ways to improve the instrument. Specifically, the current study uses a Rasch analysis to explore the response categories of the GamTest to determine whether there are valid incremental scale-steps, to examine the response pattern and goodness-of-fit between respondents and items, and to investigate the dimensionality of the instrument.

## 2. Materials and Methods

### 2.1. Procedure

The Ethical Review Board in Stockholm approved the study (dnr. 2014/545, 2017/1076-32). Data were collected using an online survey in Swedish, which was distributed to a general-population sample using a Stockholm-based polling company, SKOP, with national coverage. The authors had no way of identifying the respondents. However, SKOP used an e-mailing list so that part of the data collection can be viewed as pseudonym. The sample is the same as in [6]. The inclusion criteria were: (1) aged 18–85 years, (2) fluent in Swedish, and (3) having access to a computer. An e-mail with study information and an offer to participate was prepared by the researchers responsible for the study. The e-mail was sent to 5000 respondents that were randomly drawn from SKOP’s participant pool. After the first e-mail, three automated reminders were sent to non-responders. No compensation was given for participating in the study. The individuals that received the mail were included because the aim was to have a sample that was similar to the general Swedish population in terms of gambling habits. The survey was answered online and respondents clicked on a link in the e-mail to gain access to the survey. The respondents had to respond to each question in a section of the survey before they could move on. Because of that, there are no missing data points among the completed surveys. The respondents had to provide informed consent before answering the survey.

A total of 2376 (47.5%) respondents started answering the questionnaire, and 2257 (45.0%) answered all of the questions. Twenty-three respondents that took over two days to complete their survey were not included in the analysis. The basis for the exclusion was the assumptions that the replies were not reliable because of the long time it had taken to answer the survey, and that these respondents answered the survey on several occasions. Data from 2234 respondents were included in the analysis. The mean age of the group that did not complete the survey was 47.3 years (SD = 17.7); 45.2 years (SD = 17.5) for women and 49.4 years (SD = 17.7) for men. The mean monthly income for the attrition group was SEK 27,516 (SD = 12,893); SEK 23,682 (SD = 13,015) for women and SEK 31,512 (SD = 11,601) for men (1 USD = 9 SEK). For both, the mean age, t (2088) = 2.176, *p* = 0.03, and the mean income, t (2373) = 2.702, *p* ≤ 0.01, were significantly lower in the attrition group compared to the *n* = 2234 who completed the questionnaire. For more info, see [6].

### 2.2. Measures

The survey included the following instruments: GamTest [6,7]; PGSI [4]; Generalized Anxiety Disorder—7 Items [11], and the Patient Health Questionnaire—9 Items [12]. The latter two instruments were included to allow an examination of the relationship between the negative consequences of gambling and anxiety and depression; data from these instruments and PGSI were not included in the current study. Questions focused on gambling habits and demographics were also included. These questions were taken from the Swedish Longitudinal Gambling Study (SWELOGS) [13]. All in all, the survey contained 85 questions with 44 taken from SWELOGS. Most of the questions taken from SWELOGS focused on whether respondents used to engage in a particular form of gambling. The amount of time and money spent on a particular gambling activity was also surveyed. The frequency of the different gambling activities was also asked for. The instruments included were presented to the respondents in a random order. This was done in order to avoid possible order effects.

### 2.3. Study Sample Characteristics

The gender distribution of the 2234 respondents included in the study was 1184 (53%) men and 1048 (47%) women; 2 respondents (0.1%) responded “other.” Mean age for the total sample was 51.4 years (SD = 16.2); 50.3 years (SD = 15.5) for women and 52.4 years (SD = 16.7) for men. In total, 413 respondents (18.5%) reported negative consequences on the GamTest as a result of their gambling. These respondents constitute a sub-sample and were allocated to a group labeled “gamblers who scored over 15 points” (*n* = 413). The most common gambling activities in the sub-sample “gamblers who scored over 15 points” were in the following order: charity lotteries, lottery, sports betting, horse racing, casino (land-based and online), electronic gambling machines, bingo, and Poker (land-based and online).

The total amount gambled for in the sample was low. Hence, the sample was classified as a low-gambling population that can be seen as recreational gamblers. For more information about how the participants gambled and how they compare to the Swedish population regarding age and income, see [6].

### 2.4. GamTest

The GamTest is a self-rating scale with 15 items covering gambling habits and negative consequences due to gambling [7]. This current study used a time frame of 12 months to match that of the PGSI. However, the original version only asks about negative consequences during the past three months. For more information about the development of the GamTest, see [7].

In their study, the authors [7] found that a five-factor solution provided a good fit for the following aspects: Negative Consequences: Emotions (five questions), Negative Consequences: Money (three questions), Negative Consequences: Social (two questions), Overconsumption: Money (two questions), and Overconsumption: Time (three questions). The over-consumption factors focus on behavior; more specifically, excessive gambling behavior. For more information about the factors, see [6,7].

The item response format for our study was a rating scale ranging from 1 to 10, and employing verbal descriptors at the end points; 1 representing “Do not agree at all”, and 10 representing “Fully agree”. The range in the original version of the GamTest is from 0 to 10. The verbal descriptors are the same for when the GamTest is used in Playscan.

### 2.5. Data Analysis

To evaluate psychometric performance, a polytomous Rasch analysis was performed as per the steps described in [14], using the WINSTEPS software (3.91.0.0). In brief, a Rasch analysis involves transforming the raw scores from the GamTest into person and item equal-interval measures, employing logarithmic transformation of the odds probabilities [15]. The converted measure based on the respondents is then used to examine the person response validity (e.g., investigate item difficulty-based responses and scale precision, meaning being able to differentiate between low and high risk [16]). 

Two additions to the procedure were that an initial log-likelihood chi-squared analysis was performed to investigate if the data were suitable to perform a rating scale analysis (RSA) on, or if a partial credit model (PCM) where each item employs its own rating scale structure was more suitable [14]. Secondly, as the assumption of local independence is central in Rasch models [17], we decided to initially explore the standardized residual correlations between the included items in the GamTest. We set a criterion of r ≥ 0.5 (a shared variance of 25% or larger) as an indication of dependency between pairs of items. 

After these additional analyses, the GamTest rating scale categories were investigated using the following criteria according to [18]: (1) a minimum of 10 responses per step category, (2) for each step category the average measures should progress monotonically, and (3) values less than 2.0 for the outfit Mean Square for the step category calibrations (values around 1.0 indicate fit and values over 1.0 indicate the presence of noise deviate from model). In case the criteria were not met, the next step would be to collapse rating scale categories or delete categories, as proposed in the literature [19]. The internal structure of the GamTest was investigated by following the item goodness-of-fit statistics, both Mean Square residuals and standardized z-values, indicating how well the observed responses on the items and the expected responses match. This is based on Rasch model assertions [15]. Goodness-of-fit for items and respondents was evaluated by infit statistics. This type of statistics was chosen because it is seen as more sensitive than outfit statistics when it comes to item performance and more informative when investigating internal scale validity [15,20]. The use of the Mean Square fit statistic is preferable when examining item goodness-of-fit with polytomous data because it is less sensitive to sample size [21]. 

We chose an item goodness-of-fit set for the infit Mean Square values between 0.7 and 1.3 for the GamTest, which is a sample-size adjusted criterion for the item goodness-of-fit set for infit Mean Square values [21]. If one or more items did not provide a goodness-of-fit value that was in the set interval, the items were excluded from further analysis. The iteration process is then repeated until all items met the criterion of values between 0.7 and 1.3.

Person response validity was investigated by examining the person goodness-of-fit statistics. Infit Mean Square values >1.4 associated with a z-value >2 was the criterion used to reject person goodness-of-fit. A threat to validity is considered when more than 5% of the sample cannot succeed to demonstrate acceptable goodness-of-fit due to chance [22]. The person and item separation indices were calculated to understand the level of precision of the converted measures [23]. When investigating the range and accuracy of the individual item and person estimates, the person separation index reflects the number of statistically different groups that the instrument can identify in a given sample. The item separation index works in a similar way and indicates the amount of statistically different groups that the sample is able to identify among the items. An index above 1.5 ensures that the GamTest has the ability to differentiate at a minimum two different groups when examining the sample and the items [24]. Differential Item Functioning (DIF) analysis was used in order to investigate if the response patterns of the GamTest were stabile across age and gender. The Mantel–Haenszel statistic for polytomous scales using log-odds estimators was employed to investigate the size of DIF [25]. We set the criterion of *p* ≤ 0.01 to adjust for mass significance.

## 3. Results

### 3.1. Overall Response Pattern

In total, 413 (18.5%) of all the 2234 respondents endorsed experiencing negative consequences of their gambling. Thus, the following results are based on data from these individuals. None of the respondents had the maximum possible score of 150 and the lowest obtained score was 16, see Table 1.

### 3.2. Rating Scale Model

The log-likelihood chi-squared analysis for all of the 15 items of the GamTest resulted in the following results: 12,965.9 with approximately 13,001 degrees of freedom and *p* = 0.58, which indicates that it is possible to maintain rating scale model (RSM) instead of a PCM. The RSM provides a conceptually easier framework for the interpretation of the result because it is based on the assumption that all items have the same rating scale [26].

### 3.3. Local Independence

When exploring the standardized residual correlations between GamTest items, none of the pair exceeded the criterion of *r* > 0.5. Two item pairs (1.7%) demonstrated correlation coefficients above *r* > 0.4; items #3 and #10 (specify) *r* = 0.46, and items >#11 and #14 (specify) *r* = 0.46. Thus, we concluded that our data supported the assumption of local independence and proceeded with our analysis. 

### 3.4. Rating Scale Analysis

After the initial RSA, two scale-steps stood out in relation to the set criteria. Scale-steps 9 and 10 demonstrated a disorder in ranking based on the responses, making scale-step 10 more endorsed, and their observed count was not corrected, with scale-step 9 having a higher expected count. After merging scale-step 9 and 10 into one scale-step, a new RSA was carried out. After collapsing the scale-steps, a similar pattern of results was obtained for the new scale-steps 7 and 8, and they were collapsed in the subsequent analysis. This analysis revealed that scale-steps 4 and 5 had a similar pattern to the scale-steps merged in the previous analysis. After a last analysis, the scale-steps had an acceptable count and average count. After the analysis, a seven scale-step solution remained. 

### 3.5. Item Goodness-of-Fit

In the first iteration/analysis, four misfitting items emerged: “I sometimes borrow money to gamble”, “People close to me think I gamble too much”, “Others say I spend too much time on gambling”, and “I don’t want to tell others how much time and money I spend on gambling”, according to the item goodness-of-fit criterion. These items were, therefore, removed. The second iteration resulted in two more items exhibiting a misfit: “I sometimes feel bad when I think about my gambling” and “I spend time gambling when I should have something else.” After the third iteration, the remaining items were positioned in the recommended range of 7–13. The deleted items in the first iteration were focused on gambling in relation to others, and in the second round, the items focused on the perception that a gambler has about their gambling. After the third iteration and after removing six items (40%), the remaining nine items of the GamTest showed an acceptable item fit according to assertions made in Rasch modeling. A presentation of the frequencies and average negative consequence of gambling for the items in the revised scale is available in Table 2.

### 3.6. The Dimensionality of the Instrument

The total variance explained by the measures were 65.6% with an eigenvalue of 17.1. There were 8.1% of unexplained variance in the first contrast with an eigenvalue of 2.1 indicating a second dimension consisting of two items in the instrument besides the negative consequences of gambling. However, a value close to two is expected in the Rasch analysis. The results do not warrant changing the GamTest into two separate tests. 

### 3.7. Person Response Validity

#### 3.7.1. Person Separation

Twenty-three respondents (5.5%) had a poor goodness-of-fit for the Rasch model. A benchmark is that no more than 5% can have a poor goodness-of-fit [22]. Respondents that had minimum or maximum scores are presented in Table 1.

The person-separation index for the nine items left after the misfit analysis was 1.24 with a reliability of 0.61. The benchmark if there are two or more groups that the measure can distinguish (e.g., high and low risk) are over 1.5 with a reliability of 0.7. Our results indicate that the GamTest could not distinguish between different levels of risk. Moreover, the item-separation index (*n* = 413) was 5.39 with a reliability of 0.97, which indicates that the sample was big enough to a test whether or not there were different groups (levels of risk) in the sample.

#### 3.7.2. Endorsement of Negative Consequences of Gambling

**Figure 1:**
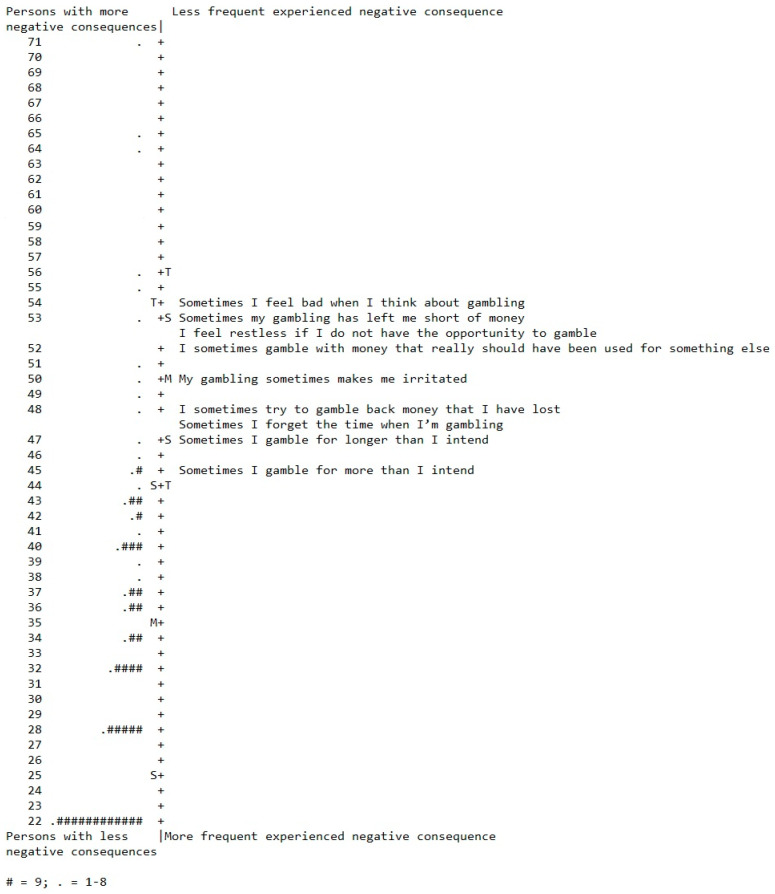
Item–person map for the final scale of the GamTest (nine items).

The person–item map is presented in Figure 1. Items indicating negative consequences of gambling more frequently experienced by the respondents are situated at the lower end of the continuum, and items indicating negative consequences of gambling less frequently experienced are situated at the higher end of the same continuum. Respondents that gamble with fewer experiences of negative effects are placed at the lower end of the continuum, and respondents with more experiences of negative effects are placed at the higher end of the continuum. The negative consequences that are endorsed the most is overconsumption in relation to money and time spent. 

#### 3.7.3. Differential Item Functioning

There were no differences in item functioning with regards to age when examining the nine items left after the goodness-of-fit analysis. However, when examining differences in item functioning for gender, three items behaved differently. These items were: “I sometimes try to gamble back money that I have lost”, which was more likely to be endorsed by men (difference between men and women was −5.72, *p* ≤ 0.01) and “I sometimes gamble with money that really should have been used for something else” (difference between men and women is 5.51, *p* ≤ 0.01) and “Sometimes I feel bad when I think of how much I have lost gambling”(difference between men and women was 1.92, *p* ≤ 0.01), which were both more likely to be endorsed by women. 

## 4. Discussion

This is the first Rasch analysis of the GamTest and one of the first studies that uses modern test theory procedures to investigate an instrument that explores the consequences, harm, or problems associated with gambling. Previous studies using a Rasch analysis have investigated PGSI and SOGS [27,28,29,30,31,32]. The advantages of using this analytic procedure are that it enables an investigation of both respondents and items simultaneously. 

A recommendation based on the RSA would be to decrease the number of scale-steps from 10 to 7. This might also result in a more equal use of all the scale-steps instead of some of the steps being underutilized. A use of all the scale might also lead to a better differentiation between levels of negative consequences in the sample. This conclusion might depend on the level of negative consequences in the total sample; that is, a high-gambling population may use all 10 scale-steps to differentiate their responses. However, defining what constitutes a low- or high-gambling population can be difficult. This paper defines low-level gambling based on the distribution of spending on gambling in [6], which presents a skewed distribution with a low degree of spending. This can be compared to the population in [7], where the level of gambling related problems (based on PGSI-scores) was higher than in [6] and can, thus, been seen as a medium-risk population. Additionally, the population in [7] was recruited at different gambling sites, which indicates a higher level of gambling seen as online gambling is associated with high risk [33]. Furthermore, when the GamTest is used in Playscan spending can also be used as a marker for low or high gambling. Playscan tracks the transactions that are made and can build a risk assessment based on that. Low-risk individuals can be presented with the instrument with revised scale-steps while medium to high-risk individuals can be presented with the non-revised version. When spending is not available, it is possible to base the decision on risk factors that the specific sample has. In a sample where there are few risk factors, the revised scale-steps can be used, while if several risk factors are present, the non-revised version administered instead. A starting point for this procedure would be to use the ranking of risk factors presented in [33]. 

PGSI only uses four scale-steps with a focus on the frequency of the behavior written out ranging from “Never” to “Always” [4]. It is possible that the GamTest also needs several scale-steps with a clear written description to accompany the scale-steps regardless of if it employs 7 or 10 scale-steps. In the current version of the GamTest, only the end points have a written description. To have a better understanding of the level of accordance with the item, a description for each scale-step or a descriptive scale-step in the middle of the scale might increase accuracy. Adding to this, the original scale has 11 scale-steps. It is plausible that the same problem will arise when using a scale that goes from 0 to 10 as when using a scale that goes from 1 to 10. 

In terms of exploring the scale’s item fit, the results demonstrated that six of the items should be excluded because they did not meet the predefined criteria. These items were: “I sometimes borrow money to gamble”, “People close to me think I gamble too much”, “Others say I spend too much time on gambling”, and “I don’t want to tell others how much time and money I spend on gambling.” When reviewing these items, they were related to social aspects of gambling, such as borrowing money from others or receiving comments from one’s surrounding with regards to gambling behavior. For a low-gambling population, these social consequences may not be relevant, resulting in misfitting items. Removing these items when targeting a low-gambling population could be one way of adjusting the scale. However, we would argue that these items should not be removed for all gamblers, as they are relevant for high-gambling populations. An item response theory approach could be used where these items are administered as a second step after the initial nine items (now preliminarily removed as a consequence of the current item fit analysis with a low-gambling sample) and only if there are indications of a high degree of gambling. A larger sample with a greater diversity in gambling may support or refute this hypothesis.

Furthermore, two more items had a poor fit, “I sometimes feel bad when I think about my gambling” and “I spend time gambling when I should have something else.” These two items focus on how a gambler might experience their emotional state after gambling, reflecting a retrospective evaluation of gambling behavior. Again, in a low-gambling population, this consequence might not be pertinent. These two items should perhaps also be presented to populations that have a higher degree of gambling. 

The remaining items that had a good fit are mainly focused on overconsumption, both in regards to time and money spent. The nine items that remain after excluding the misfit items can be seen as negative emotional states that occur when gambling too much or not having the opportunity to gamble. 

Based on the results from the person separation index, the modified nine-item version of the instrument could not differentiate between different levels of risk in the sample. This might be due to an overall low level of negative consequences due to gambling among the respondents. This is problematic when attempting to determine the level of risk for an individual who gambles and has a low risk, because there is no standard for determining when an individual has a gambling habit associated with risk.

The results showed a difference in test scores across demographics. Age did not influence the responses made across items, indicating that the instrument can be used in populations with different age groups without any systematic bias. However, due to the different functioning between men and women for some of the items, the results of the scale need to be analyzed in detail. Other instruments have shown similar tendencies. A Rasch analysis of PGSI revealed contradictory results regarding DIF when it comes to gender [27,29]. When administering the GamTest and interpreting the results, there is a need to examine what items were endorsed differently by men and women to better understand suitable feedback and interventions. Previous studies have shown that men and women have different gambling preferences, which can also be supported by the findings in this study. Women gravitate towards chance-based games (e.g., gambling machines), while men prefer skill-based games (e.g., sports betting and horse racing) [34,35,36,37,38,39,40,41]. The items that differed indicate that women might have a higher degree of social guilt when it comes to gambling. The fact that money that should be used for other purposes is spent on gambling is a potential source of guilt. Studies found that women that gamble are more prone to experience feelings of guilt and shame [42,43]. Again, this has consequences for prevention and feedback to gamblers.

### 4.1. The Results in Relation to Playscan

Previous studies suggested that the GamTest displays different psychometric properties depending on whether the population surveyed engaged in low or high levels of gambling [6,7]. Our current study shows that it is possible to make changes to the instrument, which would make it a better fit for a low-gambling population. Shortening the instrument to nine items could result in more gamblers answering essential items of the instrument. As previously stated, not all users who begin a self-test complete it [8], so these improvements may result in higher response rates. An alternative approach would be to use adaptive testing and administer the six items demonstrating a misfit, but only if the remaining nine were endorsed above a certain level. Having the individuals complete nine essential items could, in turn, lead to better risk estimates.

Furthermore, the risk assessment made on the basis of gambling patterns and the GamTest in Playscan should perhaps take into account the difference between men and women based on how they endorse the different items in the GamTest. Additionally, when using GamTest, separate norms for men and women may be required. Furthermore, given our results basing the analysis on gender may be warranted. In fact, many gambling studies do not account for gender differences, although gender-based analysis is receiving more attention in the research community at large [44].

### 4.2. Limitations

Only half of the respondents that received the e-mail answered the survey. Therefore, self-selection bias may have been introduced in the sample that answered the survey. This might be a limitation, since it can have resulted in skewed data and have produced a lower or higher degree of reported gambling-related negative consequences. In one study, the effects of selection bias in gambling research were demonstrated [45].

Another potential limitation is that the timeframe for the GamTest was changed from 3 months to 12 months. However, the authors in [46] changed the timeframes for the SOGS and the NORC Diagnostic Screen for Gambling Problems (NODS) from 12 months to 3 months, and the alteration did not have an impact on the psychometric properties of the respective instruments. The change that meant that 10 instead of 11 scale-steps was used should not have influenced the results in an extensive way, because many of the scale-steps were not used extensively. In conclusion, the alterations should not have had a significant impact on the answers provided.

### 4.3. Future Research

The findings from the current study suggest that future research should investigate the psychometric properties of a shortened GamTest with reduced scale-steps. Such a revised scale would have benefits when administered in an online gambling context. Another area that future research should address is to interview gamblers (both individuals with a high degree or a low degree of gambling) to increase the information about how individuals who gamble understand the questions and the instrument as a whole. This has been done in one study and it was found that the participants did not understand all of the items in PGSI, which in turn has implications for the administering of the scale [46]. Similar findings concerning the GamTest are plausible. Additionally, gender differences in answering the GamTest should be investigated and separate norms for men and women, both for low- and high-level gambling samples, need to be developed and used.

## 5. Conclusions

The results of the study suggest that the GamTest should be revised in order for the instrument to be suitable for administering in a low-gambling sample. Several items and scale-steps need to be revised. In line with previous research [6,47,48], this study suggests that the instruments used in gambling research may perform differently in different populations. This must be considered when using the GamTest in a general population that includes people with both low and high gambling levels.

## Figures and Tables

**Table 1:** The psychometric properties of the GamTest.

	GamTest Total Scale (15 Items)	GamTest (9 Items)
	(*n*= 413)	(*n* = 413)
Rating scale functioning	All criteria met	-
Item misfit		
1st iteration	4 items	
2nd iteration	2 items	
3rd iteration		All items met criteria
Person misfit		
*N* (%)	24 (5.5%)	
Maximum score	0	
Minimum score	58 (14.0%)	
Person separation index	-	1.24
Item separation index	-	5.39
Differential Item Functioning (DIF)	-.	No DIF for age but for gender

**Table 2:** Frequencies, Means, and Standard Deviations for the GamTest after reducing the misfit items (9 items) ^a^.

Item	Frequency (%)	M (*SD*)
Sometimes I forget the time when I’m gambling	183 (44.3%)	2.34 (2.22)
Sometimes I gamble for longer than I intend	190 (46%)	2.51 (2.33)
Sometimes I gamble more money than I intend	260 (63%)	2.75 (2.39)
I sometimes try to gamble back money that I have lost	186 (45%)	2.34 (2.24)
I sometimes gamble with money that really should have been used for something else	124 (30%)	1.93 (2.02)
Sometimes my gambling has left me short of money	90 (21.8%)	1.86 (2.14)
My gambling sometimes makes me irritated	161 (37.5%)	2.13 (2.14)
Sometimes I feel bad when I think of how much I have lost gambling	120 (29.1%)	2.04 (2.32)
I feel restless if I do not have the opportunity to gamble	116 (28.1%)	1.84 (1.85)

^a^ Based on the number of patients reporting any type of negative consequence caused by gambling, *N* = 413.

## Data Availability

The data presented in this study are available on request from the corresponding author. The data are not publicly available due to the fact that the data pseudonym.
